# Long Non-Coding RNA in Esophageal Cancer: A Review of Research Progress

**DOI:** 10.3389/pore.2022.1610140

**Published:** 2022-02-15

**Authors:** Chenbo Yang, Kuisheng Chen

**Affiliations:** ^1^ Department of Pathology, The First Affiliated Hospital of Zhengzhou University, Zhengzhou, China; ^2^ Henan Key Laboratory of Tumor Pathology, Zhengzhou University, Zhengzhou, China

**Keywords:** biomarkers, long non-coding RNA, molecular mechanism, esophageal cancer (EC), genomics

## Abstract

In recent years, there has been significant progress in the diagnosis and treatment of esophageal cancer. However, owing to the lack of early diagnosis strategies and treatment targets, the prognosis of patients with esophageal cancer remains unsatisfactory. There is an urgent need to identify novel biomarkers and treatment targets for esophageal cancer. With the development of genomics, long-chain non-coding RNAs (LncRNAs), which were once considered transcriptional “noise,” are being identified and characterized rapidly in large numbers. Recent research shows that LncRNAs are closely related to a series of steps in tumor development and play an important regulatory role in DNA replication, transcription, and post-transcriptional regulation. The abnormal expression of LncRNAs leads to tumor cell proliferation, migration, invasion, and treatment resistance. This review focuses on the latest progress in research on the abnormal expression and functional mechanisms of LncRNAs in esophageal cancer. Further, it discusses the potential applications of these findings towards achieving an early diagnosis, improving treatment efficacy, and evaluating the prognosis of esophageal cancer.

## Introduction

Esophageal cancer is one of the most common malignant tumors in the world, with the seven-highest incidence and sixth-highest mortality rate among all malignant tumors worldwide (1). Esophageal cancer is highly aggressive, which often leads to a poor prognosis. According to global cancer statistics, 1 of every 20 cancer-related deaths in 2018 was due to esophageal cancer (2, 3). There are two primary histological subtypes of esophageal cancer: esophageal squamous cell carcinoma (ESCC) and esophageal adenocarcinoma (EAC). ESCC is more common between the two, accounting for 90% of all esophageal cancer cases in China. With the advancement of diagnostic technology, including novel techniques such as narrow-band imaging with magnifying endoscopy and positron emission tomography, the accuracy of esophageal cancer diagnosis has improved greatly. In addition, advances in treatment methods, such as endoscopic surgery and neoadjuvant chemotherapy, have also significantly improved treatment efficacy. However, strategies for early diagnosis are still lacking, and most cases of esophageal cancer are diagnosed in the middle or late stages, rendering surgical treatment ineffective and leading to very poor 5-year survival rates (4). Therefore, novel biomarkers and therapeutic targets for esophageal cancer are urgently required to motivate the further development of tumor-targeted drugs and early diagnosis strategies.

**FIGURE 1:**
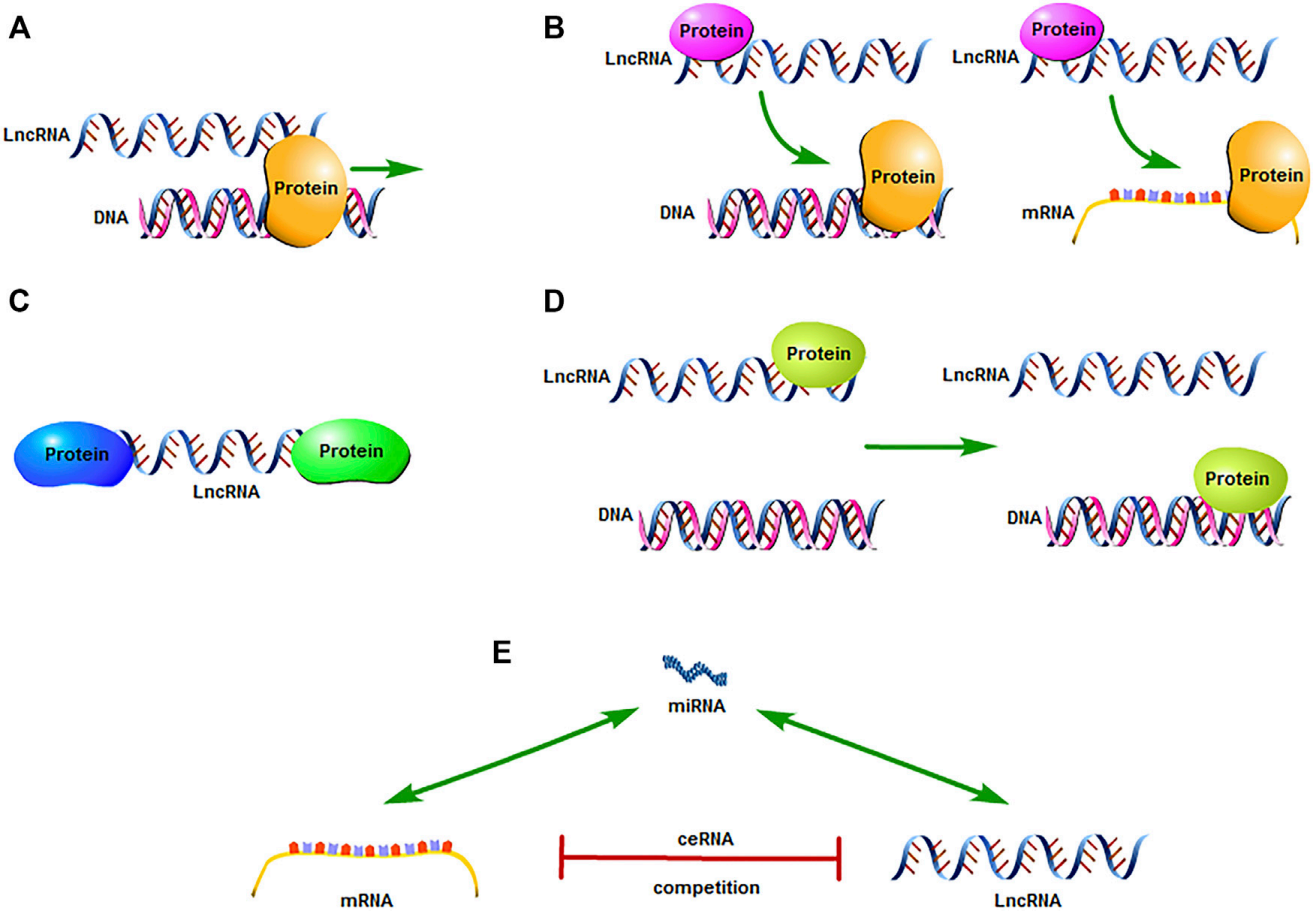
Mechanism underlying LncRNA action in tumors. **(A)** LncRNAs directly act as signaling molecule or bind to proteins to regulate downstream gene expression. **(B)** LncRNAs bind to functional proteins and affect the regulation of DNA and mRNA by protein molecules. **(C)** LncRNAs act as a “scaffold” to enable related macromolecular complexes to work together in the target area. **(D)** LncRNAs recruit functional proteins and locate and bind to the target area to exert any action. **(E)** LncRNA can be used as the “molecular sponge” of miRNA. In the form of base complementary pairing, LncRNA and target gene competitively bind miRNA, thereby reducing the silencing effect of miRNA on target gene, and further affecting the regulation of miRNA on downstream target gene.

The Human Genome Project has shown that the human genome contains about 20,000 protein-coding genes, which only account for 1.5% of all genes (5). Transcription products that are greater than 200 nucleotides in length and do not participate in protein expression are called long-chain non-coding RNAs (LncRNAs). Owing to a lack of meaningful open reading frames and protein -encoding functions, LncRNAs were once considered transcriptional “noise.” However, with the subsequent development of molecular biology, it was gradually discovered that LncRNAs regulate gene expression at different levels (epigenetic, transcriptional, and post-transcriptional) and participate in processes such as cell growth and apoptosis, protein activity regulation, and variable splicing (6, 7). For example, LncRNA-DILC binds to the IL-6 promoter, changes the gene transcription of the binding region, and inhibits the transcription of IL-6 (8) (Figure 1A); LncRNA-H19 can inhibit S-adenosine homocysteine hydrolase and increase the level of S-adenosine homocysteine. Thereby, the inhibitory effect on S-adenosylmethionine-dependent methyltransferase is enhanced, and it induces a wide range of methylation changes in the whole genome (9) (Figure 1B); LncRNA-HOTAIR can be used as a molecular scaffold to bind to histone modification complexes, combine to perform specific functions in different regions, and mediate histone methylation and demethylation (10) (Figure 1C); LncRNA-HEIH binds to histone methyltransferase EZH2, recruits EZH2 to the promoter region of the target gene, induces methylation of the promoter region of the target gene, thereby inhibiting the expression of the target gene (11) (Figure 1D); LncRNA can be used as the “molecular sponge” of miRNA. In the form of base complementary pairing, LncRNA and target gene competitively bind miRNA, thereby reducing the silencing effect of miRNA on target gene, and further affecting the regulation of miRNA on downstream target gene (Figure 1E). Studies have also found a link between the abnormal expression and dysfunction of LncRNAs and human diseases, especially malignant tumors. Therefore, LncRNAs are considered to be of great significance in tumor diagnosis, treatment, and prognostication (12).

This article reviews the latest research on the functional role and molecular mechanisms of LncRNA in esophageal cancer. Further, it discusses the significance of LncRNA in the diagnosis, treatment, and prognosis of esophageal cancer and describes the potential applications of LncRNAs as biomarkers and therapeutic targets for esophageal cancer.

## Long-Chain Non-Coding RNAs Promote the Occurrence and Development of Esophageal Cancer

**FIGURE 2:**
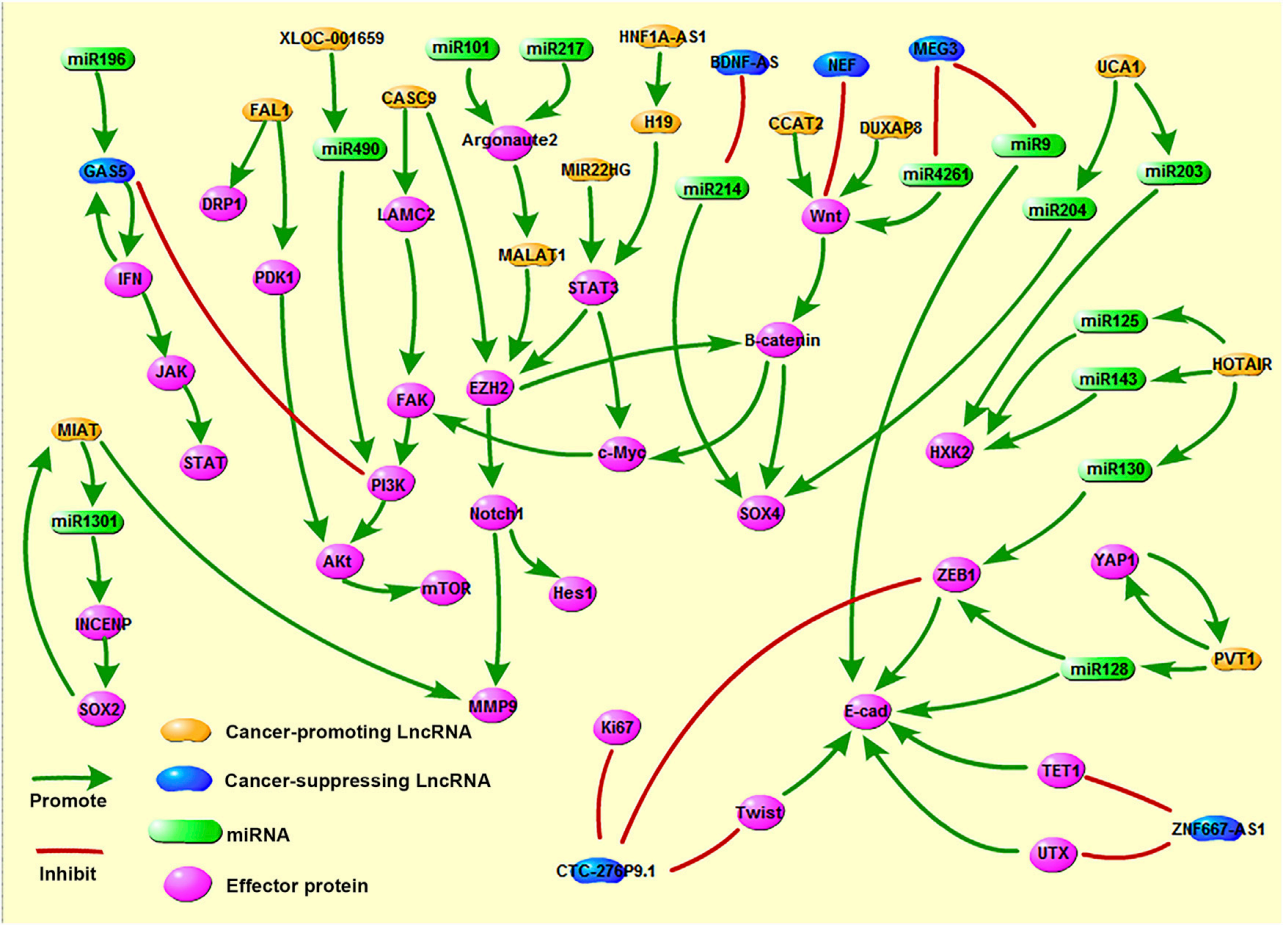
Molecular network of common LncRNAs associated with esophageal cancer.

A large number of studies have pointed out that most LncRNAs act as oncogenes in the occurrence and development of tumors (Figure 2). These LncRNAs are highly expressed in tumor tissues; promote tumor cell proliferation, migration, and invasion through various mechanisms; regulate the cell cycle; and inhibit cell apoptosis. Screening out LncRNAs with esophageal cancer-promoting effects can further elucidate the occurrence and development of tumors (Table 1). Such information can provide tools that allow the suppression of tumor growth and metastasis via gene knock-out or silencing and thereby improve prognosis (13).

**TABLE 1:** Mechanisms of common lncRNAs in regulating the progression of esophageal cancer.

LncRNA	Histological type	Effects on tumor	Mechanism	References
MALAT1	ESCC	Promote	Regulates ATM-CHK2 pathway	(15)
	ESCC	Promote	Regulates EZH2-Notch1 pathway mediates Hes1 and MMP9 proteins	(16)
	ESCC	Promote	Regulated by miR-101 and miR-217	(17)
H19	Esophageal cancer	Promote	Regulates STAT3/EZH2/β-catenin axis	(19)
	EAC	Promote	Regulated by LncRNA-HNF1A-AS1	(20)
UCA1	ESCC	Promote	Adsorbs miR-498 as ceRNA to regulate ZEB2 expression	(21)
	ESCC	Promote	Adsorbs miR-204 as ceRNA to regulate SOX4 expression	(22)
	ESCC	Promote	Adsorbs miR-203 as ceRNA to regulate HK2 expression	(23)
MIAT	ESCC	Promote	Regulates the expression of cyclin D3, CDK2, MMP2 and MMP9 proteins	(24)
	ESCC	Promote	Adsorbs miR-1301-3p as ceRNA to regulate INCENP expression	(25)
PVT1	EAC	Promote	Regulates the expression of LATS1 and YAP1 proteins	(26)
	ESCC	Promote	Regulates miR-128/ZEB1/E-cadherin axis	(27)
CCAT2	ESCC	Promote	Regulates β-catenin/WISP1 axis	(29)
	ESCC	Promote	Regulates Wnt/β-catenin pathway	(30)
CASC9	ESCC	Promote	LAMC2-mediated FAK-PI3K/Akt pathway, binds to CBP and modifies histone acetylation	(31)
	ESCC	Promote	Mediates the recruitment of EZH2 and H3K27me3, regulates the expression of PDCD4 protein	(32)
FAL1	ESCC	Promote	Regulates the expression of DRP1 protein and mitochondrial dynamics	(34)
	ESCC	Promote	Regulates the expression of PDK1 protein, activates the AKt signaling pathway	(35)
HOTAIR	ESCC	Promote	Adsorbs miR-125/miR-143 as ceRNA to regulate HK2 expression	(37)
	ESCC	Promote	Adsorbs miR-130a-5p as ceRNA to regulate ZEB1 expression	(38)
MIR22HG	EAC	Promote	Regulates STAT3/c-Myc/p-FAK pathway	(39)
XLOC-001659	ESCC	Promote	Adsorbs miR-490-5p as ceRNA to regulate PIK3CA expression	(40)
DUXAP8	ESCC	Promote	Regulates Wnt/β-catenin pathway	(41)
VESTAR	ESCC	Promote	Binds and stabilizes VEGFC mRNA	(42)
PCAT1	ESCC	Promote	Regulated by miR-326	(77)
GAS5	ESCC	Inhibit	Regulates PI3K/AKt/mTOR pathway	(44)
	ESCC	Inhibit	Regulates IFN/JAK/STAT axis	(45)
	ESCC	Inhibit	Regulated by miR-196a	(46)
NEF	ESCC	Inhibit	Regulates Wnt/β-catenin pathway	(48)
BDNF-AS	EAC	Inhibit	Adsorbs miR-214 as ceRNA	(50)
MEG3	ESCC	Inhibit	Adsorbs miR-204 as ceRNA to regulate E-cadherin and FOXO1 expression	(51)
	ESCC	Inhibit	Regulates miR-4261/Wnt/β-catenin axis	(52)
ADAMTS9-AS2	EAC	Inhibit	Regulates the expression of CDH3 proteins	(54)
ZNF667-AS1	ESCC	Inhibit	Regulates the expression of E-cadherin,and ZNF667 proteins	(55)
CTC-276P9.1	ESCC	Inhibit	Regulates the expression of Ki67, PCNA, Twist1 and ZEB1 proteins	(56)
NBAT-1	ESCC	Inhibit	Regulates the expression of PKM2 proteins	(57)
SEMA3B-AS1	ESCC	Inhibit	Regulates the expression of SEMA3B proteins	(58)
uc061hsf.1	ESCC	Inhibit	Regulated by p53	(61)

Metastasis-related lung adenocarcinoma transcript 1 (*MALAT1*) is located on human chromosome 11q13 and was first discovered in lung adenocarcinoma tissue. This gene is a marker for the metastasis and prognosis of non-small cell lung cancer (14). *MALAT1* is highly expressed in a variety of tumor tissues and is involved in tumor regulation. Hu et al. (15) showed that *MALAT1* is highly expressed in advanced esophageal cancer tissues and does not play a role in the initial stage of the tumor. Silencing the *MALAT1* gene activates the ATM-Chk2 pathway, arrests the cell cycle in the G2/M phase, and increases the rate of apoptosis. In ESCC, *MALAT1* exerts cancer-promoting effects via the dephosphorylation of the ATM, Chk2 protein. Chen et al. (16) found that in ESCC, *MALAT1* regulates the expression of Hes1 and MMP9 through the EZH2-Notch1 signaling pathway, which affects the proliferation, migration, and invasion of ESCC cells. Several LncRNAs have been found to act as ceRNAs by sponging miRNAs to reduce their inhibitory effect on their target protein-coding mRNAs. However, miRNA can also regulate the expression of LncRNA through counteraction. In ESCC, miR-101 and miR-217 act as tumor suppressor genes and down-regulate the expression of *MALAT1* through an Argonaute2-mediated pathway, thereby inhibiting cell migration and invasion (17).

*H19* is a type of commonly studied cancer-promoting LncRNA (18). Chen et al. (19) experimentally found that *H19* is highly expressed in esophageal cancer cell lines. A nude mouse xenograft model showed that *H19* knockout significantly reduced the size and weight of xenograft tumors and the protein expression of STAT3, SOX4, EZH2, and β-catenin. This demonstrates that *H19* mediates the malignant progression of esophageal cancer *via* the STAT3/EZH2/β-catenin axis *in vivo* and *in vitro*. *HNF1A-AS1* is upstream of *H19*. When *HNF1A-AS1* is inhibited, *H19* also gets inhibited, and tumor cell survival and metastasis decrease. Genes related to nucleosome and chromatin assembly in the G1/S phase of the cell cycle are also significantly affected. The molecular mechanisms *via* which *HNF1A-AS1* regulates esophageal cancer include the regulation of *H19* expression and effects on the nucleosome and chromatin assembly pathways (20).

*UCA1* has multiple molecular functions in esophageal cancer. In esophageal cancer cells, *UCA1* uses ceRNA to adsorb miR-498, causing changes in downstream ZEB2 expression and exerting cancer-promoting effects (21). *UCA1* can also act as a ceRNA to regulate the expression of the target gene SOX4. *UCA1* also binds to miR-204 to inhibit the degradation of SOX4, thereby promoting the invasion and metastasis of tumor cells (22). Studies have found that *UCA1* also promotes the expression of HXK2 by interacting with miR-203, thereby facilitating the aerobic glycolysis of tumor tissues and enhancing the Warburg effect to promote tumor cell proliferation and metastasis (23).

Myocardial infarction-associated transcript (*MIAT*), located on human chromosome 22 (19), was originally discovered as a gene associated with the risk of myocardial infarction. Zhang et al. (24) knocked out *MIAT* and found that the survival rate of esophageal cancer cells was reduced, the expression of cyclin D3 and CDK2 was reduced, and cell cycle arrest occurred in the G1 phase, leading to the inhibition of cell proliferation. Further, the levels of MMP2 and MMP9 were significantly down-regulated, and metastasis was inhibited. *MIAT* can act as a ceRNA to mediate the up-regulation of inner centromere protein (INCENP) by miR-1301-3p, forming a feedback pathway with SOX2 (*MIAT*-SOX2) and promoting the proliferation, migration, and invasion of ESCC cells (25).

The plasmacytoma variant translocation 1 (*PVT1*) gene, located on human chromosome 8q24, was the first LncRNA found in human cancer. After knocking out *PVT1* in EAC cells, the phosphorylation of LATS1 and YAP1 increased and their protein function was lost, leading to the inhibition of tumor proliferation and invasion. YAP1 knock-out also significantly inhibited the expression of *PVT1*; if YAP1 was overexpressed, the level of *PVT1* increased significantly. This indicated that LATS1 and YAP1 are downstream effectors of *PVT1*, and YAP1 has a positive feedback effect on *PVT1* (26). Hu et al. (27) suggested that *PVT1* promotes the downstream expression of ZEB1 and E-cadherin *via* miR-128, promoting ESCC progression.

Colon cancer-associated transcript 2 (*CCAT2*), which is located on chromosome 8q24 and expressed in microsatellite -stable colon cancer, was first identified as a single nucleotide polymorphism region (28). *CCAT2* silencing was found to down-regulate β-catenin and Wnt-mediated secreted protein (WISP1) expression and significantly weaken the proliferation and migration ability of tumor cells (29). Treatment of esophageal cancer cells with a Wnt inhibitor (FH535) recapitulated the effects of *CCAT2* inhibition, indicating that *CCAT2* exerts cancer-promoting effects through the Wnt/β-catenin pathway (30).

As a carcinogenic LncRNA, *CASC9* is mostly present in squamous epithelial tumors and was first discovered in ESCC (13). Liang et al. (31) suggested that *CASC9* promotes esophageal cancer metastasis through the FAK-PI3K/Akt pathway via LAMC2 and that *CASC9* stimulates the expression of LAMC2 through CBP-mediated histone acetylation to promote the invasion and metastasis of esophageal cancer. Silencing *CASC9* inhibits the growth of ESCC cells and transplanted tumors in nude mice. *CASC9* mediates the recruitment of EZH2 and H3K27me3 to the promoter region and regulates the expression of PDCD4, thereby exerting cancer-promoting effects (32).

*FAL1* is a LncRNA that has strongly been linked to malignant tumors in recent years. It is locally amplified on chromosome 1 and has a carcinogenic effect (33). Liu et al. (34) discovered a new molecular mechanism for the regulation of esophageal cancer progression. *FAL1* enhances the proliferation of ESCC cells by regulating the expression of mitochondrial division protein (DRP1) and mitochondrial dynamics. The down-regulation of *FAL1* can promote the expression of DRP1, and intracellular mitochondrial division is related to DRP1-mediated mitochondrial dysfunction. Therefore, *FAL1* silencing leads to mitochondrial dysfunction and promotes apoptosis by inhibiting mitochondrial respiration and ATP production. *FAL1* can also target PDK1 expression and activate the AKt signaling pathway, thereby promoting the proliferation of ESCC cells (35).

HOX transcribed antisense intergenic RNA (*HOTAIR*) is located in the HOXC gene cluster and has a length of 685 nucleotides. It was originally discovered as a regulator of tumor invasion and metastasis in breast cancer. At present, *HOTAIR* is a well-known LncRNA (36). Ma et al. (37) found that *HOTAIR* can effectively act as a “molecular sponge” for miR-125/miR-143, negatively regulate the expression of miR-125/miR-143, promote the expression of HK2, and ultimately promote the occurrence and development of ESCC. Wang et al. (38) found that *HOTAIR* acts as a ceRNA after binding to miR-130a-5p, regulates the expression of the downstream protein ZEB1, and promotes the epithelial–mesenchymal transition (EMT) in ESCC.

Some carcinogenic LncRNAs have been identified for the first time in esophageal cancer in recent years. Current research on these newly discovered cancer-promoting LncRNAs is not comprehensive and detailed, and further research is thus warranted. Su et al. (39) found that *MIR22HG* in EAC cells promotes tumor cell proliferation, anti-apoptotic effects, migration, and invasion through the STAT3/c-Myc/p-FAK pathway. Li et al. (40) used a LncRNA microarray analysis and found that the expression of *XLOC-001659* in esophageal cancer tissues is 20.9 times higher than that in normal esophageal tissues. *XLOC-001659* uses ceRNA to bind to miR-490-5p and promotes the occurrence and development of ESCC via the miR-490-5p/PIK3CA axis. Xu et al. (41) postulated that *DUXAP8* regulates the expression of cyclin D1 and c-Myc through the Wnt/β-catenin pathway and thereby promotes the proliferation and invasion of esophageal cancer cells. In addition to regulating downstream genes through ceRNA action, some LncRNAs also act as gene -stabilizing factors to regulate target genes, affecting protein expression and promoting cancer phenotypes. *VESTAR*, a stable VEGF-C LncRNA, can directly bind VEGF-C mRNA and maintain the stability of the mRNA structure. VEGF-C is known to be the most effective pro-lymphangiogenic factor. The high expression of *VESTAR* in ESCC tissue indicates that it may be involved in ESCC lymph node metastasis. HuR is a positive regulator of VEGF-C stability. *VESTAR* interacts with HuR to promote the binding of HuR to VEGF-C mRNA. Therefore, *VESTAR*-mediated VEGF-C mRNA stability may be an important contributor to lymph node metastasis in ESCC and could be a novel target for the diagnosis and treatment of tumor metastasis (42).

## Long-Chain Non-Coding RNAs Inhibit the Occurrence and Development of Esophageal Cancer

Although most LncRNAs are known to have cancer-promoting effects, some LncRNAs that act as tumor-suppressor genes have also been identified (Figure 2). Tumor suppressor LncRNAs inhibit tumor proliferation and migration through a variety of molecular mechanisms and also promote tumor cell apoptosis (Table 1). However, there are high levels of epigenetic regulation in tumor tissues, and the promoter region of most tumor suppressor LncRNAs are methylated. Consequently, their expression is inhibited and their tumor-suppressing action fails. Therefore, the demethylation of these promoters and up-regulation of gene expression could be a potential strategy for cancer treatment.

*GAS5* is a typical tumor suppressor LncRNA; it has been found to have a significant ability to inhibit tumor cell proliferation and promote apoptosis in lung, breast, and colorectal cancer (43). Wang et al. (44) found that *GAS5* overexpression in esophageal cancer cells significantly down-regulates the expression of PI3K and the phosphorylation levels of Akt and mTOR. The use of PI3K activators was found to reverse the inhibitory effect of *GAS5* overexpression on tumor proliferation and migration, suggesting that *GAS5* inhibits tumors by inactivating the PI3K/AKt/mTOR pathway. Huang et al. (45) discovered another anti-tumor effect of *GAS5*. They found a feedback pathway between IFN and *GAS5*, which exerts an active anti-tumor effect. When ESCC cells are cultured and treated with IFNs, the JAK-STAT signaling pathway is activated, and the expression of *GAS5* increases. This increase in *GAS5* expression in ESCC cells also promotes the interferon response in tumor cells and up-regulates the levels of IFN. *GAS5* is a positive regulator of IFN. The low expression of most tumor suppressor LncRNAs in tumor tissues is often a result of promoter methylation. However, the low expression of *GAS5* in ESCC may be a result of the effects of miR-196a. miR-196a binds to *GAS5* and down-regulates *GAS5* levels, reducing its anti-tumor effect considerably (46). miR-196a is an important oncogene. The silencing of miR-196a expression would not only inhibit its cancer-promoting effect but also increase the levels of *GAS5*, a potential target for ESCC treatment.

*NEF* is a new type of tumor suppressor LncRNA that has been discovered in hepatocellular carcinoma in recent years. NEF inhibits Wnt/β-catenin pathway to achieve tumor suppressor effect in hepatocellular carcinoma (47). Zhang et al. (48) also found that *NEF* overexpression can reduce the expression of Wnt/β-catenin pathway -related proteins in ESCC cells and thereby inhibit tumor cell proliferation, migration, and invasion. In esophageal cancer, *NEF* also participates in the inhibition of esophageal cancer by regulating the Wnt/β-catenin pathway.

Brain-derived neurotrophic factor antisense LncRNA (*BDNF-AS*) is a natural non-coding antisense RNA of the neural transcription factor BDNF, which is important for the nervous system (49). miR-214 is a crucial cancer-promoting factor. *BDNF-AS*, as the “molecular sponge” of miR-214, inhibits the proliferation, migration, and invasion of esophageal cancer cells and inhibits the EMT (50).

Maternally expressed gene 3 (*MEG3*) is located at 14q32. Studies have found that chromosomal abnormalities in this region are closely related to the occurrence and development of tumors. Dong et al. (51) found that *MEG3* acts as a ceRNA and competitively binds to miR-9 and regulates the expression of E-cadherin and FOXO1, consequently interfering with the proliferation and invasion of esophageal cancer cells. Huang et al. (52) used *in vivo* and *in vitro* experiments to show that *MEG3* can target miR-4261 and thereby block the Wnt/β-catenin signaling pathway, inhibiting tumor occurrence and development.

In recent years, researchers have found that some tumor suppressor LncRNAs in esophageal cancer directly act on downstream target proteins to regulate their expression and function. *ADAMTS9-AS2* is the antisense transcript of ADAMTS9, which acts as a tumor suppressor gene in gliomas and inhibits tumor angiogenesis. Further, it is also known to inhibit tumor formation in esophageal and nasopharyngeal cancer (53). *ADAMTS9-AS2* recruits DNMT1/DNMT3 to the promoter region of CDH3, resulting in CpG island hypomethylation and inhibiting the expression of CDH3, thereby inhibiting cancer cell function (54). *ZNF667-AS1* is located in the nucleus of esophageal cancer cells. *ZNF667-AS1* can recruit TET1 to interact with ZNF667 and E-cadherin and hydrolyze 5′-MC to 5′-HMC to further activate its expression. Moreover, *ZNF667-AS1* also changes the H3K27 methylation status in the promoter region of ZNF667 and the E-cadherin gene by interacting with UTX, thereby regulating the transcription and expression of these genes and exerting anti-tumor effects (55). *CTC-276P9.1* is also an important tumor suppressor that can inhibit the proliferation and EMT of esophageal cancer cells by directly regulating the expression of downstream proteins. Guo et al. (56) found that *CTC-276P9.1* overexpression in esophageal cancer cells can significantly reduce the expression of Ki67 and PCNA and inhibit cell proliferation. Furthermore, *CTC-276P9.1* regulates the expression of E-cadherin and Vimentin by regulating the transcription of Twist1 and ZEB1 and ultimately affects the EMT of esophageal cancer cells. Zhao et al. (57) found that the overexpression of *NBAT-1* significantly down-regulates PKM2, a key metabolic enzyme, whose disruption affects the function of tumor cells. Therefore, it has been speculated that the tumor-suppressing effect of *NBAT-1* on esophageal cancer depends on PKM2-mediated tumor glycolysis. Semaphore 3B (SEMA3B) is a tumor suppressor gene located on human chromosome 3q21.3. Its antisense long non-coding RNA (SEMA3B-AS1) is often inactivated in ESCC and loses its anti-tumor effect. CpG dinucleotide hypermethylation in the promoter region of *SEMA3B-AS1* does not affect the transcription of SEMA3B but inhibits the expression of the SEMA3B protein, indicating that *SEMA3B-AS1* may regulate SEMA3B at the post-transcriptional level (58).

p53 is the first tumor suppressor gene to be discovered, which exerts its tumor suppressor effect mainly by inducing tumor cell apoptosis and inhibiting growth (59). In subsequent studies, more and more lncRNAs have been shown to be p53 effectors, and they are involved in tumor regulation in a p53-dependent manner (60). *uc061hsf.1* is a direct transcription target of p53. *uc061hsf.1* regulates the expression of the downstream transcription factor FoxA1 and inhibits the proliferation and migration of ESCC cells, indicating that *uc061hsf.1* is a tumor suppressor LncRNA regulated by P53 (61).

## Long-Chain Non-Coding RNAs Can Indicate the Prognosis of Patients With Esophageal Cancer

Studies have confirmed that the expression of LncRNAs, both carcinogenic and tumor suppressor LncRNAs, is associated with factors such as tumor volume, pathological stage, lymph node metastasis, and histological differentiation as well as overall survival (Table 2). The differential expression of these LncRNAs in tumor tissues can help evaluate tumor progression and prognosis in patients with esophageal cancer and provide new markers for further improving esophageal cancer treatment.

**TABLE 2:** Common LncRNAs related to the pathology and prognosis of esophageal cancer.

LncRNA	Number of samples	Type of samples	Expression level	Correlation	Association with survival	References
MALAT1	100 cases	ESCC tumor tissues	Elevated	Related to TNM staging	Related to OS and DFS	(67)
	320 cases	ESCC tumor tissues	Elevated		Independent predictor of OS	(62)
CASC9	128 cases	ESCC tumor tissues	Elevated	Related to tumor staging, lymph node metastasis, and clinical staging	Independent predictor of OS and DFS	(63)
DUXAP8	78 cases	ESCC tumor tissues	Elevated	Related to tumor staging and lymph node metastasis	Related to prognosis	(41)
ZEB1-AS1	26 cases	Esophageal cancer blood	Elevated	Related to the malignant progression of tumors		(78)
	56 cases	ESCC tumor tissues	Elevated	Related to TNM staging and lymph node metastasis	Related to prognosis	(64)
H19	121 cases	ESCC tumor tissues	Elevated	Related to tumor volume and staging	Related to prognosis	(65)
	Public database	ESCC tumor tissues	Elevated		Related to prognosis	(81)
HNF1A-AS1	25 cases	EAC tumor tissues	Elevated	Related to the malignant progression of tumors		(20)
BANCR	142 cases	ESCC tumor tissues and blood	Elevated	Related to histological grade, TNM staging, lymph node metastasis	Related to OS	(66)
UCA1	100 cases	ESCC tumor tissues	Elevated	Related to TNM staging	Related to OS and DFS	(67)
	70 cases	Esophageal cancer tissues	Elevated	Related to TNM staging and lymph node metastasis		(21)
	66 cases	Esophageal cancer tissues	Elevated	Related to TNM staging and tumor differentiation	Related to prognosis	(22)
	110 cases	Esophageal cancer tissues	Elevated	Related to TNM staging, lymph node metastasis, and distant metastasis	Related to prognosis	(23)
	313 cases	ESCC exosomes in serum	Elevated	Related to tumor staging, lymph node metastasis, and clinical staging	Related to OS	(70)
POU3F3	313 cases	ESCC exosomes in serum	Elevated	Related to tumor staging, lymph node metastasis, and clinical staging	Independent predictor of OS	(70)
	78 cases	ESCC exosomes in serum	Elevated		Related to prognosis	(86)
CCAT2	33 cases	ESCC tumor tissues	Elevated		Related to OS	(29)
HOTAIR	25 cases	ESCC tumor tissues	Elevated	Related to tumor differentiation	Related to prognosis	(38)
	100 cases	ESCC tumor tissues	Elevated	Related to tumor differentiation, lymph node metastasis, and clinical staging	Independent predictor of OS	(68)
uc002yug.2	684 cases	ESCC tumor tissues	Elevated		Related to OS	(69)
PVT1	156 cases	EAC tumor tissues	Elevated	Related to histological grade and lymph node metastasis	Related to prognosis	(26)
	76 cases	ESCC tumor tissues and serum	Elevated	Related to TNM staging, lymph node metastasis, and distant metastasis	Related to OS	(27)
PCAT1	130 cases	ESCC tumor tissues	Elevated	Related to lymph node metastasis and clinical staging	Related to prognosis	(76)
	147 cases	ESCC serum	Elevated	Related to clinical staging		(77)
MEG3	143 cases	ESCC tumor tissues	Reduced	Related to TNM staging, depth of tumor invasion, lymph node metastasis, and distant metastasis	Independent predictor of OS	(51)
	28 cases	ESCC tumor tissues	Reduced		Related to prognosis	(52)
	43 cases	ESCC tumor tissues	Reduced	Related to TNM staging and lymph node metastasis	Related to prognosis	(71)
SEMA3B-AS1	138 cases	ESCC tumor tissues	Reduced	Related to TNM staging and lymph node metastasis	Related to prognosis	(58)
uc061hsf.1	34 cases	ESCC tumor tissues	Reduced	Related to lymph node metastasis and tumor differentiation	Related to prognosis	(61)

OS, total survival; DFS, disease-free survival.

*MALAT1* and *CASC9* are highly expressed in ESCC tissues and are closely related to the TNM staging. They are important independent predictors of overall and disease-free survival. These LncRNAs can act as novel indicators of tumor progression and prognosis (62, 63). *DUXAP8* and *ZEB1-AS1* are also highly expressed in ESCC tissues. Analyses have found that their expression is positively correlated with ESCC state, lymph node metastasis, and prognosis and they are potential prognostic indicators for esophageal cancer (41, 64). Li et al. (65) found that *H19* upregulation is associated with a large tumor size, high tumor stage, and short survival duration, indicating that *H19* expression could be a marker of malignant transformation and patient prognosis. Furthermore, there is a significant positive correlation between the expression of *HNF1A-AS1* and *H19*, suggesting that *HNF1A-AS1* could also be a prognostic biomarker (20). Liu et al. (66) found that the expression of *BANCR* in the ESCC patients was related to histological grade, TNM stage, lymph node metastasis and OS. After the patient’s tumor is removed, the expression level of *BANCR* returns to normal, and as the disease progresses, the expression level of *BANCR* gradually increases, which has certain potential for early diagnosis and evaluation of ESCC. Multiple studies have found that the expression of *UCA1*,*POU3F3*,*CCAT2*, *HOTAIR*, and uc002yug.2 is significantly higher in cancerous tissue than in normal adjacent tissues, and that high expression levels indicate shortened overall survival. This demonstrates that *UCA1*, *POU3F3*, *CCAT2*, *HOTAIR*, and uc002yug.2 could be important prognostic factors among patients with esophageal cancer (22, 23, 29, 67-70).

In EAC, the correlation between LncRNA and prognosis is also shown. Xu et al. (26) compared EAC tissue with Barrett’s esophagus and normal esophagus. The results showed that *PVT1* expression was up-regulated in EAC tissues, and *PVT1* expression was related to histological grade, lymph node metastasis and survival.

Some other tumor suppressor LncRNAs can also act as novel prognostic indicators in esophageal cancer. *MEG3* has obvious tumor suppressor properties in ESCC tissue, and its expression is negatively correlated with lymph node metastasis and TNM staging. Further studies have revealed that *MEG3* expression is negatively correlated with the expression of its downstream target PSAT1, high levels of which are indicative of poor patient prognosis. This suggests that *MEG3* is a potential prognostic marker that inhibits the EMT in tumor cells by inhibiting the Snail signaling pathway *via* PSAT1 (71). In line with this, Huang et al. (52) also proposed that *MEG3* expression in ESCC tissues is related to tumor volume, lymph node metastasis, and pathological staging, and *MEG3* is an independent predictor of disease-free and overall survival. The expressions of *SEMA3B-AS1* and *uc061hsf.1* are reduced in ESCC tissues and have been found to be lower in esophageal cancer tissues with lymph node metastasis or poor differentiation. This suggests that the expression of *SEMA3B-AS1* and *uc061hsf.1* is closely related to prognosis among patients with esophageal cancer (58, 61).

## Liquid Biopsy Long-Chain Non-Coding RNA Is Conducive to the Early Diagnosis of Esophageal Cancer

Even though ribonuclease levels in the blood are high, miRNA from tumor cells remains stable in serum and plasma. miRNA levels in the blood can be used as indicators of tumorigenesis. The US Food and Drug Administration has approved a series of miRNAs as tumor diagnostic indicators for clinical trials (72). Similarly, LncRNA can also be detected in human blood. Therefore, the abnormal levels of LncRNA in the blood could be a potential indicator for the early diagnosis of esophageal cancer.

Multiple LncRNAs are also differentially expressed in serum in patients with cancer. Hu et al. (27) analyzed sera from 76 ESCC patients and found that serum *PVT1* levels were related to lymph node metastasis, TNM staging, and postoperative metastasis. High levels of *PVT1* often indicate a worse overall survival, suggesting that *PVT1* could be used as a valuable serum marker for ESCC diagnosis and prognostication. *BANCR* is highly expressed in the plasma of ESCC patients. After tumor removal, plasma *BANCR* levels revert to the levels observed in healthy individuals. *BANCR* is closely related to the status of tumor activity, suggesting that it has certain potential for early diagnosis and evaluation of ESCC (66). The levels of *GAS5* and *NEF* in the cancer tissues and serum of ESCC patients are significantly lower than those in normal individuals, and the levels of *GAS5* and *NEF* also decrease with an increase in tumor stage (44, 48).

An indicator for early tumor diagnosis should not only show significant differential expression but also be stable in the blood and be easy to detect. Tong et al. (73) tested the stability and diagnostic performance of circulating LncRNA-*POU3F3* in the blood. They found that even after the serum undergoes multiple freeze–thaw cycles or is treated with acidic or alkaline solutions, *POU3F3* can still be detected and remains stable. Through receiver operating characteristic curve analysis, they found that serum *POU3F3* levels have good sensitivity and specificity in the prediction of ESCC and could be an ideal early diagnostic index for this type of malignancy.

Exosomes are the star molecules of tumor liquid biopsy. As a messenger of “intercellular communication,” they can circulate in the whole body fluid, which makes it a new type of liquid biopsy marker to attract researchers’ attention. Exosomes contain a large number of non-coding RNAs, such as microRNA (miRNA), cyclic RNA, and long-chain non-coding RNA (lncRNA). Especially during the development of tumors, exosomes can carry lncRNA that is more abundant than tumor cells. The lipid bilayer membrane structure of exosomes protects the non-coding RNA from being degraded, reduces the complexity of detecting multi-component body fluids, and increases the sensitivity and specificity of detection for low-abundance molecules (74). Therefore, LncRNA in exosomes can become an emerging biomarker.

Prostate cancer-related transcript 1 (*PCAT1*) was originally identified as an over-expressed lncRNA in prostate cancer by RNA sequencing, which can promote the progression of prostate cancer (75). In ESCC tissues, elevated *PCAT1* expression is related to tumor lymph node metastasis and clinical staging (76). More importantly, *PCAT1* was packaged into ESCC cell-derived exosomes and highly expressed in serum, ultimately promoting tumor proliferation through ceRNA interaction with miR-326 (77). *ZEB1-AS1* is derived from the ZEB1 promoter region. Exosomes have been detected in patients with esophageal cancer, and these patients have been found to show higher levels of *ZEB1-AS1* than healthy individuals. Research has also found that *ZEB1-AS1* can promote the proliferation of esophageal cancer cells by up-regulating the downstream effector protein ZEB1, demonstrating that *ZEB1-AS1* can be found in peripheral blood exosomes and may be used as a new marker for early blood-based tumor detection (78). As the most potential indicator for diagnosing ESCC, the study found that *POU3F3* also had persistently elevated expression in exosomes, and the expression level was not disturbed by experimental conditions (70).

## Regulation of Long-Chain Non-Coding RNA Expression can Enhance Tumor Sensitivity to Radiotherapy and Chemotherapy

Currently, esophageal cancer is typically treated with comprehensive strategies. Of these, neoadjuvant therapy combined with radiotherapy and chemotherapy can increase the surgical resection rate, reduce the risk of distant metastasis, and provide a therapeutic effect better than that of traditional surgical resection. However, LncRNAs affect not only the occurrence and development of esophageal cancer but also the sensitivity of tumor cells to radiotherapy and chemotherapy. Therefore, regulating the expression of LncRNAs in esophageal cancer tissues and reducing the resistance of tumor cells to radiotherapy and chemotherapy can be used as novel approaches to improve therapeutic efficacy and prevent tumor recurrence. Due to the large number of ESCC patients and the tendency to metastasize. Therefore, the current research focuses on LncRNA regulating the chemoresistance and radioresistance of ESCC.

Radiotherapy is an important step in the treatment of ESCC, and avoiding radiotherapy resistance of tumor tissue can effectively reduce recurrence and metastasis. *MALAT1* is related to the radiotherapy sensitivity of tumors. Li et al. (79) overexpressed *MALAT1* in ESCC cells and transplanted these cells to generate tumors in nude mice. After irradiation, they found that *MALAT1* overexpression enhanced tumor cell viability and reduced apoptosis. This demonstrated that *MALAT1* can inhibit the apoptosis induced by radiation and enhance the resistance of cells to radiotherapy, and *MALAT1* silencing may enhance the sensitivity of cells to radiotherapy (80). Luo et al. (81) inhibited *H19* in radiation-resistant esophageal cancer cells and found an increase in miR-22-3p expression, decrease in Wnt1 expression, and reduction in cell proliferation and migration. This suggested that *H19* can regulate the Wnt pathway via miR-22-3p and confer radiotherapy resistance. Therefore, knocking out *H19* and thereby enhancing the radiosensitivity of ESCC may be a new treatment strategy. Lin et al. (82) found that the expression of the tumor suppressor *GAS5* in radiation-sensitive cells was higher than that in radiation-resistant cells. The overexpression of *GAS5* can promote the reduction in miR-21 expression, increase the levels of RECK, and increase tumor cell apoptosis after radiotherapy. It has been suggested that *GAS5* modulates miR-21/RECK to increase the radiation sensitivity of tumor cells, and could therefore also serve as a target for improving the effect of radiotherapy.

ESCC is more sensitive to chemotherapy drugs, so chemotherapy also has good curative effect. Colon cancer-related transcript 1 (*CCAT1*) was first discovered as a carcinogen in colon cancer (83). Inhibition of the *CCAT1* gene was found to up-regulate the downstream target miR-143, reducing the expression of Ki-67 and promoting G1 arrest. miR-143 targets the expression of PLK1 and BUBR1 in ESCC cells and promotes the sensitivity of tumor cells to cisplatin drugs (84). It has been suggested that *CCAT1* can regulate the proliferation and chemotherapy resistance of esophageal cancer cells by regulating the miR-143/PLK1/BUBR1 axis. Prostate an-drogen regulated transcript 1 (*PART1*) is upregulated in gefitinib-resistant esophageal cancer cells and is associated with adverse effects of gefitinib treatment. The experimental knockout of *PART1* can promote the death of gefitinib-resistant esophageal cancer cells and reduce the resistance of ESCC to gefitinib (85). *POU3F3* is not only of great significance in the early diagnosis of ESCC, but also can guide the selection of chemotherapy drugs. Tumor cell-derived exosomes contain *POU3F3*, which induces the transformation of normal fibroblasts to tumor-associated fibroblasts, increases the level of IL-6 in the tumor microenvironment, and promotes cisplatin resistance in ESCC cells(86).

## Applying Public Databases to Discover More Potentially Valuable Long-Chain Non-Coding RNAs

The methods to study the regulatory function of LncRNA mainly include traditional biological experimental methods and modern computational methods of bioinformatics. Traditional biological experimental methods to identify the function of LncRNAs, although the results are accurate and reliable, have problems such as long experimental cycle time and high cost. With the continuous development of high-throughput sequencing technology, more and more LncRNAs have been discovered, and the functions of a large number of LncRNAs need to be clarified. Traditional biological experimental methods are obviously incompetent, and fast and efficient computational methods must be used to conduct data mining of the discovered relationship between LncRNAs and human diseases, and then infer the function of LncRNAs (87).

LncRNADisease: In 2012, Chen et al. (88) collected the relationship between LncRNAs in the regulation of human diseases reported in the PubMed database and developed the first database of LncRNAs in the regulation of human diseases. LncRNADisease gives the PubMed hyperlink of the original article for each LncRNA-disease association data, and annotates the detailed information of the LncRNA-disease association, including genomic information, sequence information, dysfunction type, etc.

Lnc2Cancer: In 2015, Ning et al. (89) established an experimentally supported LncRNA database that specifically collects LncRNA-cancer associations. Each association data in the database includes the name of LncRNA and cancer, LncRNA sequence and location information, LncRNA expression pattern, experimental technique, LncRNA functional description, PubMed database hyperlink and other annotation information.

LncRNADisease2.0 is an updated version of LncRNADisease, which was established by Bao et al. (90) in 2018. Compared to the first version of LncRNADisease, LncRNADisease 2.0 has significant improvements. For example, transcriptional regulatory relationships between LncRNAs, mRNAs, and miRNAs are provided; disease names are mapped to the MeSH database, and quantitative confidence scores are provided for each LncRNA-disease association. LncRNADisease 2.0 is one of the most comprehensive databases for collecting LncRNA-disease association data.

## Summary and Outlook

As our understanding of LncRNA increases, our awareness of the key roles LncRNAs play in the functioning of various cells under normal and disease states is also increasing. LncRNAs participate in gene regulation via a variety of molecular mechanisms. For example, some LncRNAs act as a “molecular sponge” to regulate downstream miRNAs, whereas others act as epigenetic regulators that affect the expression of effector proteins or as protein chaperones to affect protein function. Studies have confirmed that there are several LncRNAs in tumor tissues and they perform various functions, and most studies have reported the specific molecular mechanisms underlying these processes. LncRNAs are promising new biomarkers that can assist in early disease diagnosis, improve the curative effect of treatment, and predict patient prognosis. Further, the discovered LncRNA regulatory networks and molecular pathways could provide potential avenues for targeted tumor therapy.

However, some LncRNAs participate in multiple molecular pathways in tumors. One example is *HOTAIR*, which is involved in both the miR-125/miR-143/HK2 axis and the miR-130a-5p/ZEB1 pathway. It has been confirmed that signaling molecules usually interact with each other and function together. A single LncRNA is not sufficient to drive cell signal transduction, and accordingly, a single signaling molecule may not work effectively. Therefore, our understanding of LncRNAs is still at the nascent stages. It is necessary to discover more LncRNAs related to signaling pathways, further characterize how LncRNAs and signaling molecules work co-operatively, and map the multi-factor regulatory networks that include both LncRNA and miRNA. This will help in the application of LncRNAs as biomarkers for early tumor diagnosis and prognostic assessment and targets for precise treatment.

## References

[1] Fan J Liu Z Mao X Tong X Zhang T Suo C Global Trends in the Incidence and Mortality of Esophageal Cancer from 1990 to 2017. Cancer Med (2020) 9:e03338. 10.1002/cam4.3338 PMC7520289 32750217

[2] Bray F Ferlay J Soerjomataram I Siegel RL Torre LA Jemal A. Global Cancer Statistics 2018: GLOBOCAN Estimates of Incidence and Mortality Worldwide for 36 Cancers in 185 Countries. CA: A Cancer J Clinicians (2018) 68(6):394–424. 10.3322/caac.21492 30207593

[3] Wong MCS Hamilton W Whiteman DC Jiang JY Qiao Y Fung FDH Global Incidence and Mortality of Oesophageal Cancer and Their Correlation with Socioeconomic Indicators Temporal Patterns and Trends in 41 Countries. Sci Rep (2018) 8(1):4522. 10.1038/s41598-018-19819-8 29540708 PMC5852053

[4] Ohashi S Miyamoto Si. Kikuchi O Goto T Amanuma Y Muto M. Recent Advances from Basic and Clinical Studies of Esophageal Squamous Cell Carcinoma. Gastroenterology (2015) 149(7):1700–15. 10.1053/j.gastro.2015.08.054 26376349

[5] Maher B. ENCODE: The Human Encyclopaedia. Nature (2012) 489(7414):46–8. 10.1038/489046a 22962707

[6] Lee JT. Epigenetic Regulation by Long Noncoding RNAs. Science (2012) 338(6113):1435–9. 10.1126/science.1231776 23239728

[7] Yoon J-H Kim J Gorospe M. Long Noncoding RNA Turnover. Biochimie (2015) 117:15–21. 10.1016/j.biochi.2015.03.001 25769416 PMC4565787

[8] Wang X Sun W Shen W Xia M Chen C Xiang D Long Non-coding RNA DILC Regulates Liver Cancer Stem Cells via IL-6/STAT3 axis. J Hepatol (2016) 64(6):1283–94. 10.1016/j.jhep.2016.01.019 26812074

[9] Zhou J Yang L Zhong T Mueller M Men Y Zhang N H19 lncRNA Alters DNA Methylation Genome Wide by Regulating S-Adenosylhomocysteine Hydrolase. Nat Commun (2015) 6:10221. 10.1038/ncomms10221 26687445 PMC4703905

[10] Wu Y Zhang L Wang Y Li H Ren X Wei F Long Noncoding RNA HOTAIR Involvement in Cancer. Tumor Biol (2014) 35(10):9531–8. 10.1007/s13277-014-2523-7 25168368

[11] Yang F Zhang L Huo X-s. Yuan J-h. Xu D Yuan S-x. Long Noncoding RNA High Expression in Hepatocellular Carcinoma Facilitates Tumor Growth through Enhancer of Zeste Homolog 2 in Humans. Hepatology (2011) 54(5):1679–89. 10.1002/hep.24563 21769904

[12] Thin KZ Liu X Feng X Raveendran S Tu JC LncRNA-Dancr. LncRNA-DANCR: A Valuable Cancer Related Long Non-coding RNA for Human Cancers. Pathol - Res Pract (2018) 214(6):801–5. 10.1016/j.prp.2018.04.003 29728310

[13] Qi Y Song C Zhang J Guo C Yuan C. Oncogenic LncRNA CASC9 in Cancer Progression. Curr Pharm Des (2021) 27(4):575–82. 10.2174/1381612826666200917150130 32940174

[14] Ji P Diederichs S Wang W Böing S Metzger R Schneider PM MALAT-1, a Novel Noncoding RNA, and Thymosin β4 Predict Metastasis and Survival in Early-Stage Non-small Cell Lung Cancer. Oncogene (2003) 22(39):8031–41. 10.1038/sj.onc.1206928 12970751

[15] Hu L Wu Y Tan D Meng H Wang K Bai Y Up-regulation of Long Noncoding RNA MALAT1 Contributes to Proliferation and Metastasis in Esophageal Squamous Cell Carcinoma. J Exp Clin Cancer Res (2015) 34:7. 10.1186/s13046-015-0123-z 25613496 PMC4322446

[16] Chen M Xia Z Chen C Hu W Yuan Y. LncRNA MALAT1 Promotes Epithelial-To-Mesenchymal Transition of Esophageal Cancer through Ezh2-Notch1 Signaling Pathway. Anti-Cancer Drugs (2018) 29(8):767–73. 10.1097/cad.0000000000000645 29916899

[17] Wang X Li M Wang Z Han S Tang X Ge Y Silencing of Long Noncoding RNA MALAT1 by miR-101 and miR-217 Inhibits Proliferation, Migration, and Invasion of Esophageal Squamous Cell Carcinoma Cells. J Biol Chem (2015) 290(7):3925–35. 10.1074/jbc.m114.596866 25538231 PMC4326802

[18] Tan D Wu Y Hu L He P Xiong G Bai Y Long Noncoding RNA H19 Is Up-Regulated in Esophageal Squamous Cell Carcinoma and Promotes Cell Proliferation and Metastasis. Dis Esophagus (2017) 30(1):1–9. 10.1111/dote.12481 27247022

[19] Chen M-J Deng J Chen C Hu W Yuan Y-C Xia Z-K. LncRNA H19 Promotes Epithelial Mesenchymal Transition and Metastasis of Esophageal Cancer via STAT3/EZH2 axis. Int J Biochem Cel Biol (2019) 113:27–36. 10.1016/j.biocel.2019.05.011 31102664

[20] Yang X Song JH Cheng Y Wu W Bhagat T Yu Y Long Non-coding RNAHNF1A-AS1regulates Proliferation and Migration in Oesophageal Adenocarcinoma Cells. Gut (2014) 63(6):881–90. 10.1136/gutjnl-2013-305266 24000294 PMC4612639

[21] Wang P Liu X Han G Dai S Ni Q Xiao S Downregulated lncRNA UCA1 Acts as ceRNA to Adsorb microRNA-498 to Repress Proliferation, Invasion and Epithelial Mesenchymal Transition of Esophageal Cancer Cells by Decreasing ZEB2 Expression. Cell Cycle (2019) 18(18):2359–76. 10.1080/15384101.2019.1648959 31387451 PMC6738578

[22] Jiao C Song Z Chen J Zhong J Cai W Tian S lncRNA-UCA1 Enhances Cell Proliferation through Functioning as a ceRNA of Sox4 in Esophageal Cancer. Oncol Rep (2016) 36(5):2960–6. 10.3892/or.2016.5121 27667646

[23] Liu HE Shi HH Luo XJ. Upregulated Long Noncoding RNA UCA1 Enhances Warburg Effect via miR-203/HK2 Axis in Esophagal Cancer. J Oncol (2020) 2020:8847687. 10.1155/2020/8847687 33204264 PMC7657677

[24] Zhang W Chen Q Lei C. lncRNA MIAT Promotes Cell Invasion and Migration in Esophageal Cancer. Exp Ther Med (2020) 19(5):3267–74. 10.3892/etm.2020.8588 32266022 PMC7132222

[25] Zhang C Xie L Fu Y Yang J Cui Y. lncRNA MIAT Promotes Esophageal Squamous Cell Carcinoma Progression by Regulating miR-1301-3p/INCENP axis and Interacting with SOX2. J Cel Physiol (2020) 235(11):7933–44. 10.1002/jcp.29448 31943174

[26] Xu Y Li Y Jin J Han G Sun C Pizzi MP LncRNA PVT1 Up-Regulation Is a Poor Prognosticator and Serves as a Therapeutic Target in Esophageal Adenocarcinoma. Mol Cancer (2019) 18(1):141. 10.1186/s12943-019-1064-5 31601234 PMC6785865

[27] Hu J Gao W. Long Noncoding RNA PVT1 Promotes Tumour Progression via the miR-128/ZEB1 axis and Predicts Poor Prognosis in Esophageal Cancer. Clin Res Hepatol Gastroenterol (2021) 45(4):101701. 10.1016/j.clinre.2021.101701 33848670

[28] Kasagi Y Oki E Ando K Ito S Iguchi T Sugiyama M The Expression of CCAT2, a Novel Long Noncoding RNA Transcript, and Rs6983267 Single-Nucleotide Polymorphism Genotypes in Colorectal Cancers. Oncology (2017) 92(1):48–54. 10.1159/000452143 27875818

[29] Yang C Li F Zhou W Huang J. Knockdown of Long Non-coding RNA CCAT2 Suppresses Growth and Metastasis of Esophageal Squamous Cell Carcinoma by Inhibiting the β-catenin/WISP1 Signaling Pathway. J Int Med Res (2021) 49(5):3000605211019938. 10.1177/03000605211019938 34057837 PMC8753796

[30] Wang X Wang X. Long Non-coding RNA colon Cancer-Associated Transcript 2 May Promote Esophageal Cancer Growth and Metastasis by Regulating the Wnt Signaling Pathway. Oncol Lett (2019) 18(2):1745–54. 10.3892/ol.2019.10488 31423241 PMC6607085

[31] Liang Y Chen X Wu Y Li J Zhang S Wang K LncRNA CASC9 Promotes Esophageal Squamous Cell Carcinoma Metastasis through Upregulating LAMC2 Expression by Interacting with the CREB-Binding Protein. Cell Death Differ (2018) 25(11):1980–95. 10.1038/s41418-018-0084-9 29511340 PMC6219493

[32] Wu Y Hu L Liang Y Li J Wang K Chen X Up-regulation of lncRNA CASC9 Promotes Esophageal Squamous Cell Carcinoma Growth by Negatively Regulating PDCD4 Expression through EZH2. Mol Cancer (2017) 16(1):150. 10.1186/s12943-017-0715-7 28854977 PMC5577767

[33] Zhong X Hu X Zhang L. Oncogenic Long Noncoding RNA FAL1 in Human Cancer. Mol Cell Oncol (2015) 2(2):e977154. 10.4161/23723556.2014.977154 27308441 PMC4905162

[34] Liu T Wang Z Zhou R Liang W. Focally Amplified lncRNA on Chromosome 1 Regulates Apoptosis of Esophageal Cancer Cells via DRP1 and Mitochondrial Dynamics. IUBMB Life (2019) 71(2):254–60. 10.1002/iub.1971 30501006

[35] Liang XS Sun Y Liu T. Long Non-coding RNA FAL1 Regulated Cell Proliferation through Akt Pathway via Targeting PDK1 in Esophageal Cancer Cells. Eur Rev Med Pharmacol Sci (2018) 22(16):5214–22. 10.26355/eurrev_201808_15719 30178844

[36] Rinn JL Kertesz M Wang JK Squazzo SL Xu X Brugmann SA Functional Demarcation of Active and Silent Chromatin Domains in Human HOX Loci by Noncoding RNAs. Cell (2007) 129(7):1311–23. 10.1016/j.cell.2007.05.022 17604720 PMC2084369

[37] Ma J Fan Y Feng T Chen F Xu Z Li S HOTAIR Regulates HK2 Expression by Binding Endogenous miR-125 and miR-143 in Oesophageal Squamous Cell Carcinoma Progression. Oncotarget (2017) 8(49):86410–22. 10.18632/oncotarget.21195 29156804 PMC5689694

[38] Wang W Wu D He X Hu X Hu C Shen Z CCL18-induced HOTAIR Upregulation Promotes Malignant Progression in Esophageal Squamous Cell Carcinoma through the miR-130a-5p-ZEB1 axis. Cancer Lett (2019) 460:18–28. 10.1016/j.canlet.2019.06.009 31207321

[39] Su W Guo C Wang L Wang Z Yang X Niu F LncRNA MIR22HG Abrogation Inhibits Proliferation and Induces Apoptosis in Esophageal Adenocarcinoma Cells via Activation of the STAT3/c-Myc/FAK Signaling. Aging (2019) 11(13):4587–96. 10.18632/aging.102071 31291201 PMC6660029

[40] Li F-Z Zang W-Q. Knockdown of lncRNAXLOC_001659 Inhibits Proliferation and Invasion of Esophageal Squamous Cell Carcinoma Cells. World J Gastroenterol (2019) 25(42):6299–310. 10.3748/wjg.v25.i42.6299 31754291 PMC6861847

[41] Xu LJ Yu XJ Wei B Hui HX Sun Y Dai J Long Non-coding RNA DUXAP8 Regulates Proliferation and Invasion of Esophageal Squamous Cell Cancer. Eur Rev Med Pharmacol Sci (2018) 22(9):2646–52. 10.26355/eurrev_201805_14959 29771416

[42] Wang Y Zhang W Liu W Huang L Wang Y Li D Long Noncoding RNA VESTAR Regulates Lymphangiogenesis and Lymph Node Metastasis of Esophageal Squamous Cell Carcinoma by Enhancing VEGFC mRNA Stability. Cancer Res (2021) 81(12):3187–99. 10.1158/0008-5472.can-20-1713 33771898

[43] Ma C Shi X Zhu Q Li Q Liu Y Yao Y The Growth Arrest-specific Transcript 5 (GAS5): a Pivotal Tumor Suppressor Long Noncoding RNA in Human Cancers. Tumor Biol (2016) 37(2):1437–44. 10.1007/s13277-015-4521-9 26634743

[44] Wang G Sun J Zhao H Li H. Long Non-coding RNA (lncRNA) Growth Arrest Specific 5 (GAS5) Suppresses Esophageal Squamous Cell Carcinoma Cell Proliferation and Migration by Inactivating Phosphatidylinositol 3-kinase (PI3K)/AKT/Mammalian Target of Rapamycin (mTOR) Signaling Pathway. Med Sci Monit (2018) 24:7689–96. 10.12659/msm.910867 30368517 PMC6216480

[45] Huang J Li Y Lu Z Che Y Sun S Mao S Long Non-coding RNA GAS5 Is Induced by Interferons and Plays an Antitumor Role in Esophageal Squamous Cell Carcinoma. Cancer Med (2018) 7:3157–67. 10.1002/cam4.1524 PMC6051207 29745062

[46] Wang K Li J Xiong G He G Guan X Yang K Negative Regulation of lncRNA GAS5 by miR-196a Inhibits Esophageal Squamous Cell Carcinoma Growth. Biochem Biophysical Res Commun (2018) 495(1):1151–7. 10.1016/j.bbrc.2017.11.119 29170131

[47] Liang W-C Ren J-L Wong C-W Chan S-O Waye MM-Y Fu W-M LncRNA-NEF Antagonized Epithelial to Mesenchymal Transition and Cancer Metastasis via Cis-Regulating FOXA2 and Inactivating Wnt/β-Catenin Signaling. Oncogene (2018) 37(11):1445–56. 10.1038/s41388-017-0041-y 29311643

[48] Zhang J Hu SL Qiao CH Ye JF Li M Ma HM LncRNA-NEF Inhibits Proliferation, Migration and Invasion of Esophageal Squamous-Cell Carcinoma Cells by Inactivating Wnt/β-Catenin Pathway. Eur Rev Med Pharmacol Sci (2018) 22(20):6824–31. 10.26355/eurrev_201810_16150 30402846

[49] Modarresi F Faghihi MA Lopez-Toledano MA Fatemi RP Magistri M Brothers SP Inhibition of Natural Antisense Transcripts *In Vivo* Results in Gene-specific Transcriptional Upregulation. Nat Biotechnol (2012) 30(5):453–9. 10.1038/nbt.2158 22446693 PMC4144683

[50] Zhao H Diao C Wang X Xie Y Liu Y Gao X LncRNA BDNF-AS Inhibits Proliferation, Migration, Invasion and EMT in Oesophageal Cancer Cells by Targeting miR-214. J Cel Mol Med (2018) 22:3729–39. 10.1111/jcmm.13558 PMC6050505 29896888

[51] Dong Z Zhang A Liu S Lu F Guo Y Zhang G Aberrant Methylation-Mediated Silencing of lncRNA MEG3 Functions as a ceRNA in Esophageal Cancer. Mol Cancer Res (2017) 15(7):800–10. 10.1158/1541-7786.mcr-16-0385 28539329

[52] Huang Z-L Chen R-P Zhou X-T Zhan H-L Hu M-M Liu B Long Non-coding RNA MEG3 Induces Cell Apoptosis in Esophageal Cancer through Endoplasmic Reticulum Stress. Oncol Rep (2017) 37(5):3093–9. 10.3892/or.2017.5568 28405686

[53] Yao J Zhou B Zhang J Geng P Liu K Zhu Y A New Tumor Suppressor LncRNA ADAMTS9-AS2 Is Regulated by DNMT1 and Inhibits Migration of Glioma Cells. Tumor Biol (2014) 35(8):7935–44. 10.1007/s13277-014-1949-2 24833086

[54] Liu D Wu K Yang Y Zhu D Zhang C Zhao S. Long Noncoding RNA ADAMTS9-AS2 Suppresses the Progression of Esophageal Cancer by Mediating CDH3 Promoter Methylation. Mol Carcinog (2020) 59(1):32–44. 10.1002/mc.23126 31621118

[55] Dong Z Li S Wu X Niu Y Liang X Yang L Aberrant Hypermethylation-Mediated Downregulation of Antisense lncRNA ZNF667-AS1 and its Sense Gene ZNF667 Correlate with Progression and Prognosis of Esophageal Squamous Cell Carcinoma. Cell Death Dis (2019) 10(12):930. 10.1038/s41419-019-2171-3 31804468 PMC6895126

[56] Guo W Liu S Dong Z Guo Y Ding C Shen S Aberrant Methylation-Mediated Silencing of lncRNA CTC-276P9.1 Is Associated with Malignant Progression of Esophageal Squamous Cell Carcinoma. Clin Exp Metastasis (2018) 35(1-2):53–68. 10.1007/s10585-018-9881-2 29524086

[57] Zhao B Cao P Hu S Li F Kong K Zu Y. LncRNA-NBAT-1 Modulates Esophageal Cancer Proliferation via PKM2. Am J Transl Res (2019) 11(9):5978–87. 31632565 PMC6789236

[58] Dong Z Liang X Wu X Kang X Guo Y Shen S Promoter Hypermethylation-Mediated Downregulation of Tumor Suppressor Gene SEMA3B and lncRNA SEMA3B-AS1 Correlates with Progression and Prognosis of Esophageal Squamous Cell Carcinoma. Clin Exp Metastasis (2019) 36(3):225–41. 10.1007/s10585-019-09964-3 30915595

[59] Bieging KT Mello SS Attardi LD. Unravelling Mechanisms of P53-Mediated Tumour Suppression. Nat Rev Cancer (2014) 14(5):359–70. 10.1038/nrc3711 24739573 PMC4049238

[60] Zhang A Xu M Mo Y-Y. Role of the lncRNA-P53 Regulatory Network in Cancer. J Mol Cel Biol (2014) 6(3):181–91. 10.1093/jmcb/mju013 PMC4034727 24721780

[61] Yao J Zhang H Li H Qian R Liu P Huang J. P53-regulated lncRNA uc061hsf.1 Inhibits Cell Proliferation and Metastasis in Human Esophageal Squamous Cell Cancer. IUBMB Life (2020) 72(3):401–12. 10.1002/iub.2196 31743955

[62] Wang C Zhang Q Hu Y Zhu J Yang J. Emerging Role of Long Non-coding RNA MALAT1 in Predicting Clinical Outcomes of Patients with Digestive System Malignancies: A Meta-Analysis. Oncol Lett (2019) 17(2):2159–70. 10.3892/ol.2018.9875 30719108 PMC6350192

[63] Gao GD Liu XY Lin Y Liu HF Zhang GJ. LncRNA CASC9 Promotes Tumorigenesis by Affecting EMT and Predicts Poor Prognosis in Esophageal Squamous Cell Cancer. Eur Rev Med Pharmacol Sci (2018) 22(2):422–9. 10.26355/eurrev_201801_14191 29424900

[64] Zhao Y Wang N Zhang X Liu H Yang S. LncRNA ZEB1-AS1 Down-Regulation Suppresses the Proliferation and Invasion by Inhibiting ZEB1 Expression in Oesophageal Squamous Cell Carcinoma. J Cel Mol Med (2019) 23(12):8206–18. 10.1111/jcmm.14692 PMC6850966 31638344

[65] Li X Yang H Wang J Li X Fan Z Zhao J High Level of lncRNA H19 Expression Is Associated with Shorter Survival in Esophageal Squamous Cell Cancer Patients. Pathol - Res Pract (2019) 215(11):152638. 10.1016/j.prp.2019.152638 31551175

[66] Liu Z Yang T Xu Z Cao X. Upregulation of the Long Non-coding RNA BANCR Correlates with Tumor Progression and Poor Prognosis in Esophageal Squamous Cell Carcinoma. Biomed Pharmacother (2016) 82:406–12. 10.1016/j.biopha.2016.05.014 27470379

[67] Kang K Huang YH Li HP Guo SM. Expression of UCA1 and MALAT1 Long-Chain Non-coding RNAs in Esophageal Squamous Cell Carcinoma Tissues Is Predictive of Patient Prognosis. Arch Med Sci (2018) 14(4):752–9. 10.5114/aoms.2018.73713 30002691 PMC6040126

[68] Li X Wu Z Mei Q Li X Guo M Fu X Long Non-coding RNA HOTAIR, a Driver of Malignancy, Predicts Negative Prognosis and Exhibits Oncogenic Activity in Oesophageal Squamous Cell Carcinoma. Br J Cancer (2013) 109(8):2266–78. 10.1038/bjc.2013.548 24022190 PMC3798955

[69] Wu H Zheng J Deng J Zhang L Li N Li W LincRNA-uc002yug.2 Involves in Alternative Splicing of RUNX1 and Serves as a Predictor for Esophageal Cancer and Prognosis. Oncogene (2015) 34(36):4723–34. 10.1038/onc.2014.400 25486427

[70] Yan S Du L Jiang X Duan W Li J Xie Y Evaluation of Serum Exosomal lncRNAs as Diagnostic and Prognostic Biomarkers for Esophageal Squamous Cell Carcinoma. Cancer Manag Res (2020) 12:9753–63. 10.2147/cmar.s250971 33116835 PMC7548224

[71] Li M Liu L Zhang W Zhan H Chen R Feng J Long Non-coding RNA MEG3 Suppresses Epithelial-To-Mesenchymal Transition by Inhibiting the PSAT1-dependent GSK-3β/Snail Signaling Pathway in Esophageal Squamous Cell Carcinoma. Oncol Rep (2020) 44(5):2130–42. 10.3892/or.2020.7754 32901893 PMC7550985

[72] Zhang J Zhao H Gao Y Zhang W. Secretory miRNAs as Novel Cancer Biomarkers. Biochim Biophys Acta (Bba) - Rev Cancer (2012) 1826(1):32–43. 10.1016/j.bbcan.2012.03.001 22440944

[73] Tong Y-S Wang X-W Zhou X-L Liu Z-H Yang T-X Shi W-H Identification of the Long Non-coding RNA POU3F3 in Plasma as a Novel Biomarker for Diagnosis of Esophageal Squamous Cell Carcinoma. Mol Cancer (2015) 14:3. 10.1186/1476-4598-14-3 25608466 PMC4631113

[74] Shao H Im H Castro CM Breakefield X Weissleder R Lee H. New Technologies for Analysis of Extracellular Vesicles. Chem Rev (2018) 118(4):1917–50. 10.1021/acs.chemrev.7b00534 29384376 PMC6029891

[75] Prensner JR Iyer MK Balbin OA Dhanasekaran SM Cao Q Brenner JC Transcriptome Sequencing across a Prostate Cancer Cohort Identifies PCAT-1, an Unannotated lincRNA Implicated in Disease Progression. Nat Biotechnol (2011) 29(8):742–9. 10.1038/nbt.1914 21804560 PMC3152676

[76] Shi W-h. Wu Q-q. Li S-q. Yang T-x. Liu Z-h. Tong Y-s. Upregulation of the Long Noncoding RNA PCAT-1 Correlates with Advanced Clinical Stage and Poor Prognosis in Esophageal Squamous Carcinoma. Tumor Biol (2015) 36(4):2501–7. 10.1007/s13277-014-2863-3 25731728

[77] Huang L Wang Y Chen J Wang Y Zhao Y Wang Y Long Noncoding RNA PCAT1, a Novel Serum-Based Biomarker, Enhances Cell Growth by Sponging miR-326 in Oesophageal Squamous Cell Carcinoma. Cel Death Dis (2019) 10(7):513. 10.1038/s41419-019-1745-4 PMC6609620 31273188

[78] Zhang YG Zhou MW Bai L Han RY Lv K Wang Z. Extracellular Vesicles Promote Esophageal Cancer Progression by Delivering lncZEB1-AS1 between Cells. Eur Rev Med Pharmacol Sci (2018) 22(9):2662–70. 10.26355/eurrev_201805_14962 29771414

[79] Li Z Zhou Y Tu B Bu Y Liu A Kong J. Long Noncoding RNA MALAT1 Affects the Efficacy of Radiotherapy for Esophageal Squamous Cell Carcinoma by Regulating Cks1 Expression. J Oral Pathol Med (2017) 46(8):583–90. 10.1111/jop.12538 27935117

[80] Farooqi A Legaki E Gazouli M Rinaldi S Berardi R. MALAT1 as a Versatile Regulator of Cancer: Overview of the Updates from Predatory Role as Competitive Endogenous RNA to Mechanistic Insights. Curr Cancer Drug Targets (2020) 21:192–202. 10.2174/1568009620999200730183110 32748748

[81] Luo W Liu W Yao J Zhu W Zhang H Sheng Q Downregulation of H19 Decreases the Radioresistance in Esophageal Squamous Cell Carcinoma Cells. OncoTargets Ther (2019) 12:4779–88. 10.2147/ott.s203235 PMC6592057 31417277

[82] Lin J Liu Z Liao S Li E Wu X Zeng W. Elevation of Long Non-coding RNA GAS5 and Knockdown of microRNA-21 Up-Regulate RECK Expression to Enhance Esophageal Squamous Cell Carcinoma Cell Radio-Sensitivity after Radiotherapy. Genomics (2020) 112(3):2173–85. 10.1016/j.ygeno.2019.12.013 31866421

[83] Nissan A Stojadinovic A Mitrani-Rosenbaum S Halle D Grinbaum R Roistacher M Colon Cancer Associated Transcript-1: a Novel RNA Expressed in Malignant and Pre-malignant Human Tissues. Int J Cancer (2012) 130(7):1598–606. 10.1002/ijc.26170 21547902

[84] Hu M Zhang Q Tian XH Wang JL Niu YX Li G. lncRNA CCAT1 Is a Biomarker for the Proliferation and Drug Resistance of Esophageal Cancer via the miR-143/PLK1/BUBR1 axis. Mol Carcinog (2019) 58(12):2207–17. 10.1002/mc.23109 31544294

[85] Kang M Ren M Li Y Fu Y Deng M Li C. Exosome-mediated Transfer of lncRNA PART1 Induces Gefitinib Resistance in Esophageal Squamous Cell Carcinoma via Functioning as a Competing Endogenous RNA. J Exp Clin Cancer Res (2018) 37(1):171. 10.1186/s13046-018-0845-9 30049286 PMC6063009

[86] Tong Y Yang L Yu C Zhu W Zhou X Xiong Y Tumor-Secreted Exosomal lncRNA POU3F3 Promotes Cisplatin Resistance in ESCC by Inducing Fibroblast Differentiation into CAFs. Mol Ther - Oncolytics (2020) 18:1–13. 10.1016/j.omto.2020.05.014 32637576 PMC7321817

[87] Moran VA Perera RJ Khalil AM. Emerging Functional and Mechanistic Paradigms of Mammalian Long Non-coding RNAs. Nucleic Acids Res (2012) 40(14):6391–400. 10.1093/nar/gks296 22492512 PMC3413108

[88] Chen G Wang Z Wang D Qiu C Liu M Chen X LncRNADisease: a Database for Long-Non-Coding RNA-Associated Diseases. Nucleic Acids Res (2013) 41(Database issue):D983–6. 10.1093/nar/gks1099 23175614 PMC3531173

[89] Ning S Zhang J Wang P Zhi H Wang J Liu Y Lnc2Cancer: a Manually Curated Database of Experimentally Supported lncRNAs Associated with Various Human Cancers. Nucleic Acids Res (2016) 44(D1):D980–D985. 10.1093/nar/gkv1094 26481356 PMC4702799

[90] Bao Z Yang Z Huang Z Zhou Y Cui Q Dong D. LncRNADisease 2.0: an Updated Database of Long Non-coding RNA-Associated Diseases. Nucleic Acids Res (2019) 47(D1):D1034–d1037. 10.1093/nar/gky905 30285109 PMC6324086

